# Genetic and morphological analyses of *Gracilaria firma* and *G*. *changii* (Gracilariaceae, Rhodophyta), the commercially important agarophytes in western Pacific

**DOI:** 10.1371/journal.pone.0182176

**Published:** 2017-07-31

**Authors:** Poh-Kheng Ng, Showe-Mei Lin, Phaik-Eem Lim, Anicia Q. Hurtado, Siew-Moi Phang, Yoon-Yen Yow, Zhongmin Sun

**Affiliations:** 1 Institute of Marine Biology, National Taiwan Ocean University, Keelung, Taiwan, R.O.C; 2 Institute of Ocean and Earth Sciences, University of Malaya, Kuala Lumpur, Malaysia; 3 Integrated Services for the Development of Aquaculture and Fisheries (ISDA) Inc., Tabuc Suba, Iloilo City, Philippines; 4 Department of Biological Sciences, School of Science and Technology, Sunway University, Selangor, Malaysia; 5 Laboratory of Marine Organism Taxonomy and Phylogeny, Institute of Oceanology, Chinese Academy of Sciences, Qingdao, China; National Cheng Kung University, TAIWAN

## Abstract

Many studies classifying *Gracilaria* species for the exploitation of agarophytes and the development of the agar industry were conducted before the prevalence of molecular tools, resulting in the description of many species based solely on their morphology. *Gracilaria firma* and *G*. *changii* are among the commercially important agarophytes from the western Pacific; both feature branches with basal constrictions that taper toward acute apices. In this study, we contrasted the morpho-anatomical circumscriptions of the two traditionally described species with molecular data from samples that included representatives of *G*. *changii* collected from its type locality. Concerted molecular analyses using the *rbc*L and *cox*1 gene sequences, coupled with morphological observations of the collections from the western Pacific, revealed no inherent differences to support the treatment of the two entities as distinct taxa. We propose merging *G*. *changii* (a later synonym) into *G*. *firma* and recognize *G*. *firma* based on thallus branches with abrupt basal constrictions that gradually taper toward acute (or sometimes broken) apices, cystocarps consisting of small gonimoblast cells and inconspicuous multinucleate tubular nutritive cells issuing from gonimoblasts extending into the inner pericarp at the cystocarp floor, as well as deep spermatangial conceptacles of the *verrucosa*-type. The validation of specimens under different names as a single genetic species is useful to allow communication and knowledge transfer among groups from different fields. This study also revealed considerably low number of haplotypes and nucleotide diversity with apparent phylogeographic patterns for *G*. *firma* in the region. Populations from the Philippines and Taiwan were divergent from each other as well as from the populations from Malaysia, Thailand, Singapore and Vietnam. Establishment of baseline data on the genetic diversity of this commercially important agarophyte is relevant in the context of cultivation, as limited genetic diversity may jeopardize the potential for its genetic improvement over time.

## Introduction

Following the discovery in the 1950s that good-quality agar can be produced from *Gracilaria* Greville by pre-treating the agarophyte with alkali before agar extraction, the food grade agar industry experienced rapid expansion that resulted in the uncontrolled harvesting of various *Gracilaria* species from natural stands [1]. Concerns about limited supplies from natural resources due to overharvesting to meet the increasing demand of the agar industry has led to the development of the cultivation of *Gracilaria* worldwide, including in the Asia-Pacific region. The market values of dried seaweed and extracted colloids are affected by the various properties of agars as well as the proportion of agarose to agar fractions, which are species-specific [2]. Many species of *Gracilaria* had been described solely based on their morphology for the exploitation of agarophytes and the development of the agar industry before the prevalent use of molecular tools to aid in taxonomic classification and species identification.

Among the commercially important agarophytes, *Gracilaria firma* Chang & Xia and *G*. *changii* (Xia & Abbott) Abbott, Zhang & Xia are traditionally described in the western Pacific. *Gracilaria firma* was established [3] to accommodate the Chinese materials that feature thalli with a firm texture, branches with basal constrictions that taper toward acute apices, gradual transition in cell size from cortex to medulla, cystocarps with small gonimoblast cells and an absence of nutritive filaments (also as traversing filaments [4], absorbing filaments [5], and tubular nutritive cells [6]), as well as spermatangia borne in elliptical cavities (as the *verrucosa*-type spermatangial conceptacles). It is distributed in tropical and subtropical regions of the western Pacific, including China, Vietnam, Thailand, Malaysia and the Philippines [7]. *Gracilaria firma* has been cultivated on a commercial scale in Taiwan [8], Vietnam [9] and the Philippines [10]. Its simple and variable morphology has caused *G*. *firma* to be misidentified in many instances. Agarophytes resembling *G*. *verrucosa* (Hudson) Papenfuss, *G*. *blodgettii* Harvey and *G*. *fisheri* (Xia & Abbott) Abbott, Zhang & Xia in gross morphology found along the coastline of Vietnam were identified as *G*. *firma*. They share similar *rbc*L sequences and show reproductive anatomy that is consistent with the description for *G*. *firma* [11]. *Gracilaria tenuistipitata* var. *liui* was noted as a misnomer for *G*. *firma* in Taiwan [12]. The Taiwanese species is closely related to the Vietnamese *G*. *firma* (unpublished data) and different from *G*. *tenuistipitata* var. *liui* (NC_006137), based on molecular analyses of the *rbc*L sequences [11]. Apart from that, *G*. *firma* was also easily mistaken for *G*. *changii* because the two are only readily distinguished by the type of spermatangial conceptacles [13]. The latter was first described as *Polycavernosa changii* Xia & Abbott [5] based on Malaysian specimens characterized by branches with abrupt constrictions at the base and tapering gradually toward apices, abrupt transition of cell size from cortex to medulla, cystocarps with two-layered pericarp, small gonimoblast cells and scarcely present basal nutritive filaments, as well as spermatangia borne in multicavitied conceptacles (as the *Polycavernosa*-type spermatangial conceptacles). *Gracilaria changii* was reported to have a narrower distribution in Malaysia, Thailand, Myanmar and the Philippines compared to *G*. *firma* [7]. Agar of superior quality had been extracted from *G*. *changii*, rendering it a good candidate for potential commercialization [14]. The traditional taxonomic stance of *G*. *firma* and *G*. *changii* has not been verified using molecular tools, despite their overlapping regional distribution (see [7]) as well as their similar morphology.

In this study, we employed a molecular-assisted approach to delineate the *G*. *firma/G*. *changii* complex by conducting molecular analyses and detailed morpho-anatomical observations on the collections from the Southeast Asian region and Taiwan. Images of the type specimens for *G*. *firma*, *G*. *changii* and *G*. *fisheri* were sought from herbaria for morphological comparison with our collections. Analyses of specimens that included representatives of *G*. *changii* collected from type localities suggested the conspecificity of *G*. *changii* and *G*. *firma*. The oldest validly published name, *G*. *firma*, is proposed to be adopted for the species considered as *G*. *changii*. The genetic diversity of *G*. *firma* in the western Pacific was also inferred. The validation of specimens passing under the name of *G*. *changii* as *G*. *firma* would deconvolute a small part of the *Gracilaria* taxonomy and somewhat ease the transfer of cultivation knowledge across the region.

## Materials and methods

### Ethics statement

The samples are not endangered or protected species. No specific permits were required for this study, as the specimens were not collected from any national parks or protected areas. Consent was granted from the owner for specimen collection from cultivation farm.

### Taxon sampling and sample processing

**Fig 1:**
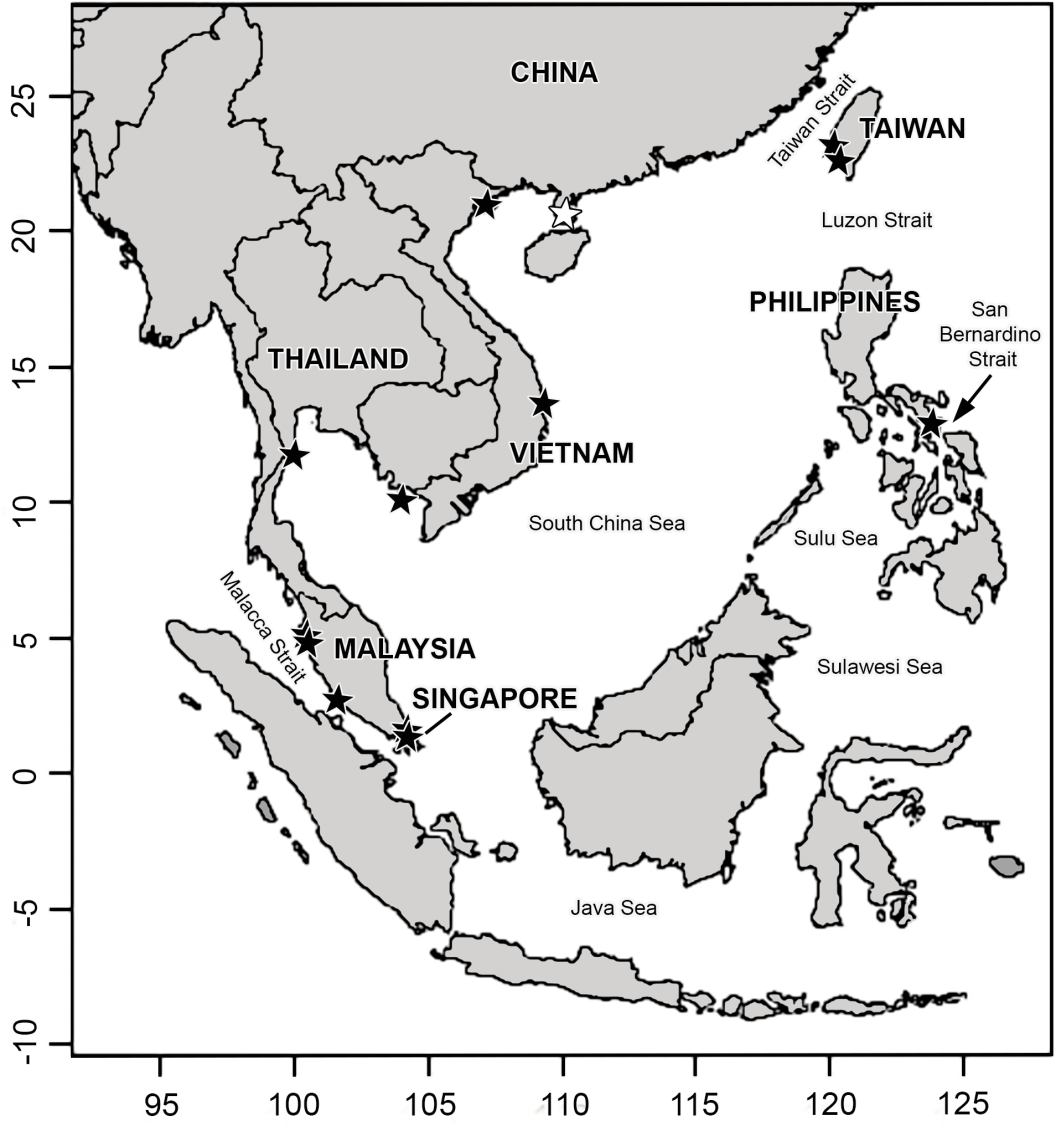
Map showing distribution of *Gracilaria firma*. The white star denotes the type locality at Xuwen County in the Guangdong Province of China; black stars denote the general collection locations and do not correspond to sample size.

**Fig 2:**
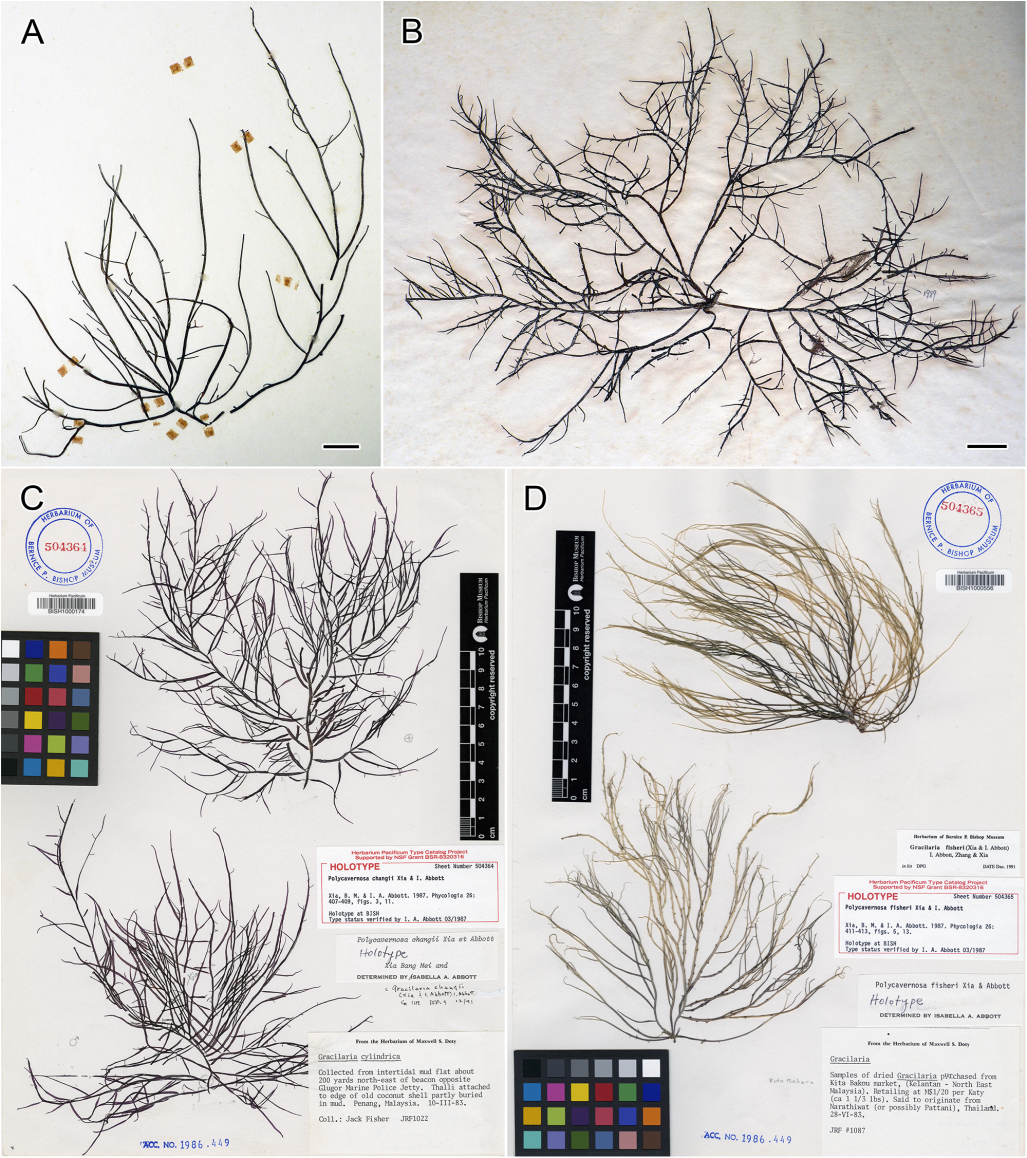
*Gracilaria firma* Chang & Xia, *G*. *changii* (Xia & Abbott) Abbott, Zhang & Xia and *G*. *fisheri* (Xia & Abbott) Abbott, Zhang & Xia. **(A)** Holotype of *G*. *firma*, AST 66–540, tetrasporophyte, collected on 2 April 1966 from Xindi, Xuwen County, Guangdong Province, China. Scale bar = 1 cm. **(B)** Isotype of *G*. *firma*, AST 66-540a, cystocarpic plant, collected on 2 April 1966 from Xindi, Xuwen County, Guangdong Province, China. Scale bar = 1 cm. **(C)** Holotype of *G*. *changii*, Fisher # 1022, tetrasporic and male plants, collected on 10 March 1983 from Middle Bank, Penang, Malaysia. **(D)** Holotype of *G*. *fisheri*, Fisher # 1087, cystocarpic plant, collected on 28 June 1983 near Pattani, Thailand.

Specimens collected from Malaysia (n = 5), Singapore (n = 4), Thailand (n = 3), Vietnam (n = 4), Taiwan (n = 11) and the Philippines (n = 17) were identified as *G*. *changii*, *G*. *fisheri* and *G*. *firma* (Fig 1). Species identification was based on reference to regional literature [2–3, 5, 12, 15–16] as well as the samples’ geographical provenance, with detailed morpho-anatomical analyses whenever possible. Scanned images of the type specimens for *G*. *firma*, *G*. *changii* and *G*. *fisheri* (Fig 2) were obtained from the Herbaria of the Institute of Oceanology, Academia Sinica [now Chinese Academia of Sciences] (AST), and the B.P. Bishop Museum, Honolulu, Hawaii (BISH), for morphological comparison with the specimens examined in this study. The isotype sample preserved in formalin was too old and fragmented for making any sections to obtain useful information. Attempt to collect samples of *G*. *firma* from the type locality, Xuwen County, Guangdong Province of China was unsuccessful. Representative *G*. *changii* samples were collected from the type locality in Penang, Malaysia (PSM10466 and PSM10481). Pulau Korea Besar is the local name given to an islet situated east of Gelugor in the Middle Bank, Penang, which is representative of the location where *G*. *changii* was typified. All specimens were dried and preserved as vouchers, either as a herbarium press or in silica gel from which a subsample of material was taken for DNA sequencing, and a subsample for some of the specimens were preserved in 5% formalin in seawater for morphological study. Collection information and GenBank accession numbers for the *rbc*L and *cox*1 gene sequence of the specimens examined in this study are listed in S1 Table, with the samples used for morphological analyses indicated by voucher number in boldface. Hand sections were stained with Wittmann’s aceto-iron-hematoxylin-chloral hydrate [17] and mounted in 50% Hoyer’s mounting medium [18]. Photomicrographs were taken on an Olympus BX51 microscope with a Q-imaging digital camera (Burnaby, BC, Canada), and habit views were taken on a Nikon D300 camera (Tokyo, Japan).

Genomic DNA was extracted from silica gel-preserved samples or herbarium vouchers using an *i*-genomic Plant DNA Extraction Mini Kit (iNtRON Biotechnology Inc., South Korea) according to the manufacturer’s recommendations. PCR amplifications of each marker were carried out using primers F7/RrbcS start or F7/R1434 [19–21] for *rbc*L and COXI43F/COXI1549R [22] for *cox*1, with the PCR conditions described in [23]. PCR products were cleaned using the LaboPass Gel & PCR purification kit (Cosmo Genetech, Seoul, South Korea) and sequenced commercially in both directions using the amplification primers. DNA of the isotype of *G*. *firma* was also extracted, but it failed to amplify.

### Molecular analyses

Overlapping bidirectional reads of each sample for each marker were edited and assembled into a consensus sequence using ChromasPro v.1.5 (Technelysium Pty Ltd., Queensland, Australia). Electropherograms were manually examined and checked for the occurrence of multiple peaks upon encountering ambiguous nucleotides in the sequence reads. Both *rbc*L and *cox*1 datasets were aligned using ClustalX v.2.0 [24] with the default gap extension/opening parameters, and the alignments were trimmed with BioEdit v.7.0.5.3 [25]. Additional sequences of Gracilariaceae, particularly those of *Gracilaria sensu lato* nested within *Hydropuntia* Montagne complex [26], were downloaded from GenBank and included in the phylogenetic analyses along with the sequences newly generated from this study. A large number of the *cox*1 sequences for Gracilariaceae available in GenBank are only about 600–700 base pairs (bp) to serve as molecular barcode for species identification. Alignments for the *rbc*L and *cox*1 genes used in the phylogenetic analyses consisted of 1070 and 1118 bp, respectively, after trimming. Identical sequences were removed from the alignment and tree reconstructions were made using one haplotype by country. Trimmed alignments used for phylogenetic analyses are available from the authors upon request.

Phylogenetic reconstruction for each dataset was inferred using maximum likelihood (ML) and Bayesian analysis. Maximum likelihood tree searches were implemented in PhyML 3.0 online execution version [27], based on the GTR+G+I model automatically selected by the program using the Akaike Information Criterion for the *rbc*L and *cox*1 datasets. Branch support was evaluated using the SH-like approximate Likelihood Ratio Test (aLRT) implemented in PhyML with 1000 bootstrap replicates. Bayesian inference (BI) was conducted with MrBayes v.3.2.6 [28]. The best-fitting substitution model, GTR+G, was selected for both datasets based on the Bayesian Information Criterion implemented in Kakusan v.3.0.2009.03.08 [29]. Bayesian analyses were initiated with a random starting tree and two parallel runs, each of which consisted of running one cold chain and three hot chains of Markov chain Monte Carlo (MCMC) iterations for 2×10^6^ generations. The trees in each chain were sampled every 200^th^ generation. The convergence of the two MCMC runs to a stationary distribution was determined by looking at the standard deviation of split frequencies (which were always less than 0.01) and by the convergence of the parameter values in the two independent runs. The first 200 trees were discarded as burn-in, and the remaining trees were used to calculate a 50% majority rule tree and to determine the posterior probabilities for both datasets. The *rbc*L and *cox*1 phylogenies were rooted with *Gracilariopsis* species, based on the phylogenetic relationships inferred from global searches for the Gracilariaceae [26, 30]. The absolute distances and uncorrected pairwise distances for the *rbc*L and *cox*1 sequences of the specimens analyzed in this study and related reference taxa were computed with MEGA6 [31].

For haplotype parsimony network analysis, the *rbc*L sequences from GenBank of *G*. *changii* from Thailand (JQ026049) and *G*. ‘*blodgettii*’ from China (JQ407695) were aligned with those newly generated in this study. While the newly generated *cox*1 sequences formed a well-supported monophyletic clade with several GenBank entries for *G*. *changii* and *G*. ‘*blodgettii*’ from Malaysia, the Philippines and China, network analysis for *cox*1 was performed only with the sequences generated in this study and published sequences of *G*. *changii* [32–33], which encompassed specimens collected from the type locality in Middle Bank, Penang, Malaysia. The *cox*1 sequences for *G*. ‘*blodgettii*’ and *G*. *changii* from China (JQ407591) and the Philippines (KX017516, KT779908, KT779928 and KX017515) were not included in the network analysis, as the rather short consensus (less than 200 bp after trimming) that can result from alignment was not informative enough. The *rbc*L and *cox*1 alignments were trimmed to 1070 and 834 bp, respectively. A parsimony network was constructed for each alignment in TCS v.1.2.1 [34] using a default connection limit of 95%. Estimates of haplotype and nucleotide diversity based on *rbc*L and *cox*1 were calculated for the overall dataset, as well as populations from each country considered in the haplotype parsimony network analyses except for China which was represented only by a single *rbc*L sequence, by using DnaSP v.5.10.01 [35].

## Results

### Molecular analyses

**Fig 3:**
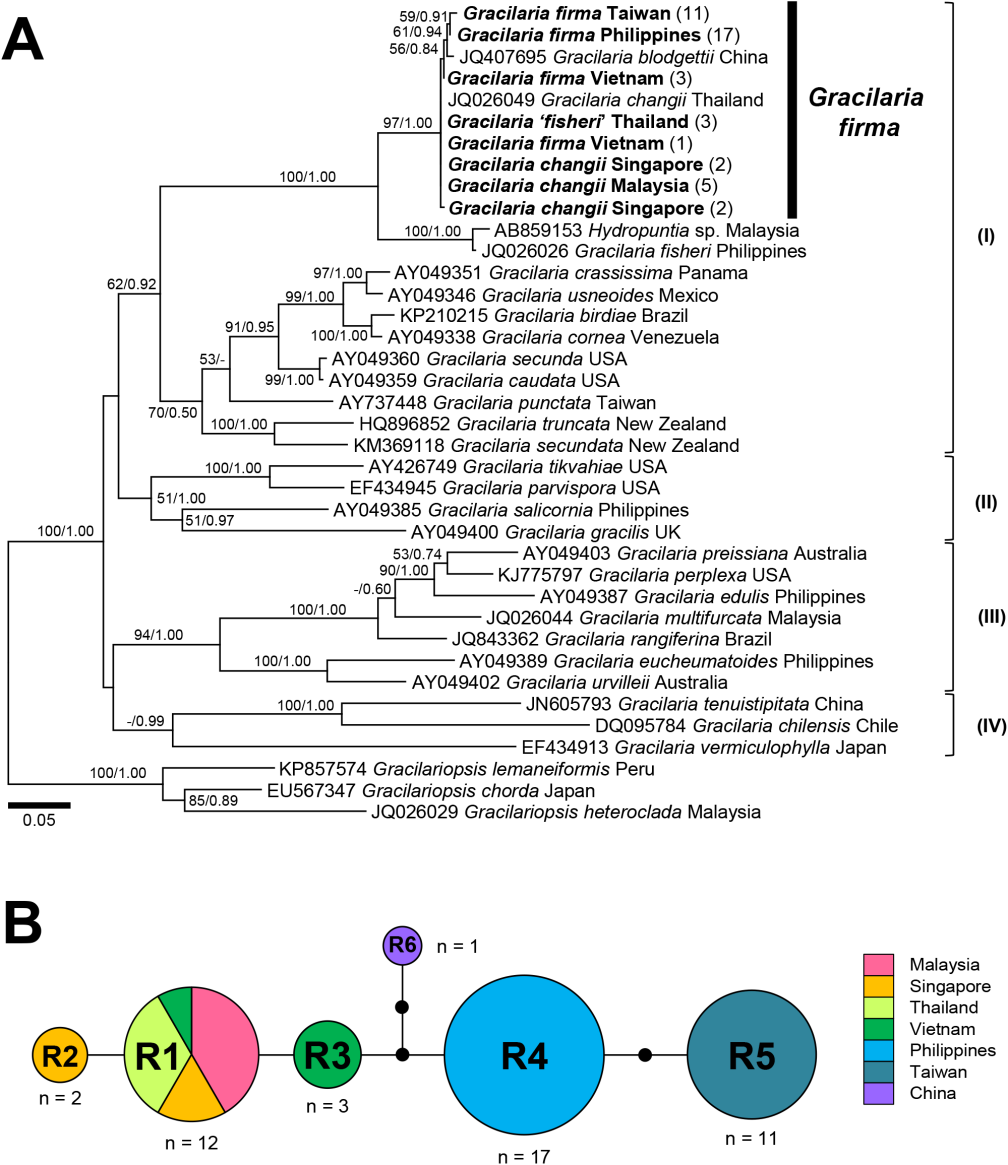
Phylogeny and haplotype network for *Gracilaria firma* based on the *rbc*L gene. **(A)** Phylogeny of *Gracilaria* inferred from the *rbc*L gene alignment consisting of 1070 base pairs (bp) using the ML method (-lnL = 6707.49073). Numbers at the nodes denote ML bootstrap support and Bayesian posterior probabilities. Dashes denote ML bootstrap support or Bayesian posterior probabilities of less than 50% or 0.50, respectively. The numbers in parentheses after the taxon denote the number of specimens with identical sequences from each country. **(B)** Haplotype network for *G*. *firma* based on 1070 bp of *rbc*L. Circle sizes are proportional to haplotype frequency. Lines connecting the haplotypes represent single base pair mutations. Small black circles represent undetected or hypothetical haplotypes. Haplotypes are colored according to the geographic origin as shown in the key.

***rbc*L gene.** The *rbc*L gene produced an unambiguously aligned dataset for 38 sequences which included 1070 bp, of which 370 were variable and 291 were parsimony-informative. Both phylogenetic inference methods (ML and BI) recovered a fully supported ingroup of *Gracilaria* (Fig 3A) made up of four distinct clades (I, II, III and IV) with poor to moderate ML bootstrap support (BS) but high Bayesian posterior probability (PP), each corresponding to ‘Atlantic’ *Hydropuntia* (subgroup III), *Gracilaria sensu stricto* (subgroups IV, V and VIII), ‘Pacific’ *Hydropuntia* (subgroup II) and a putative new genus (subgroup I) identified in [26]. The topology derived using the ML method was incongruent with that of BI. Clade I formed a poorly supported sister clade with Clade II in the phylogeny inferred using ML analysis, but it formed a weakly supported monophyletic assemblage of *Hydropuntia* (PP = 0.87) with Clade III in the phylogeny derived from BI (data not shown). The samples identified as *G*. *firma* from Taiwan, Vietnam and the Philippines, *G*. *changii* from Malaysia and Singapore, and *G*. ‘*fisheri*’ from Thailand formed a strongly supported monophyletic clade (BS = 97% / PP = 1.00), along with reference sequences of *G*. ‘*blodgettii*’ from China (JQ407695) and *G*. *changii* from Thailand (JQ026049) in Clade I. They differed from each other by a maximum of 0.6% pairwise differences, suggesting conspecificity of these taxa. These specimens were most closely related to an unnamed Malaysian ‘*Hydropuntia*’ species (AB859153) and a *G*. ‘*fisheri*’ sample from the Philippines (JQ026026), to which they differed by 4.0–4.8%. The interspecific divergence of *G*. *firma*/*G*.*changii*/*G*. ‘*fisheri*’ (from Thailand) analyzed in this study and other congeners within Clade I ranged from 7.4% to 9.3%.

Six haplotypes were inferred from the partial *rbc*L gene and they were connected by a maximum of six mutational steps (Fig 3B). Apart from the samples from Singapore and Vietnam, which were represented by two haplotypes, samples from other countries consisted of only a single haplotype: R1 for the Malaysian and Thai samples, R4 for the Philippine samples and R5 for the Taiwanese samples (Fig 3B, S1 Table). The haplotype R1 was represented by *G*. *changii* from Malaysia and Singapore, *G*. *firma* from Vietnam and *G*. ‘*fisheri*’ from Thailand, whereas haplotypes R2 and R3 were exclusively represented by *G*. *changii* from Singapore and *G*. *firma* from Vietnam, respectively. Haplotypes R2 and R3 differed from haplotype R1 only by a single base pair. Haplotype R5 was more closely connected to haplotype R4, with two mutational steps, than to the haplotypes R1-R3 from the mainland-bound countries, with four to six mutational steps. Representing *G*. ‘*blodgettii*’ from China, haplotype R6 was connected to haplotypes R3 and R4 by three mutational steps each (Fig 3B, Table 1). The overall haplotype and nucleotide diversities of the *Gracilaria* populations in the western Pacific region examined in this study based on the *rbc*L gene were 0.748 ± 0.033 and 0.00216 ± 0.00016, respectively. While samples from Vietnam and Singapore were both represented by two haplotypes, the former recorded a higher haplotype and nucleotide diversity (0.667 and 0.00062) compared to the latter (0.500 and 0.00047).

**Table 1:** Variation sites in DNA sequences of *Gracilaria firma* for haplotypes based on the *rbc*L gene.

Haplotypes	Variation sites in 1070-base pair alignment							
	9	312	408	521	691	827	942	1034
R1	C	C	A	T	A	G	T	T
R2	C	T	A	T	A	G	T	T
R3	C	C	A	T	A	G	C	T
R4	T	C	A	C	A	G	C	T
R5	T	C	A	C	A	A	C	C
R6	C	C	G	C	G	G	C	T

**Fig 4:**
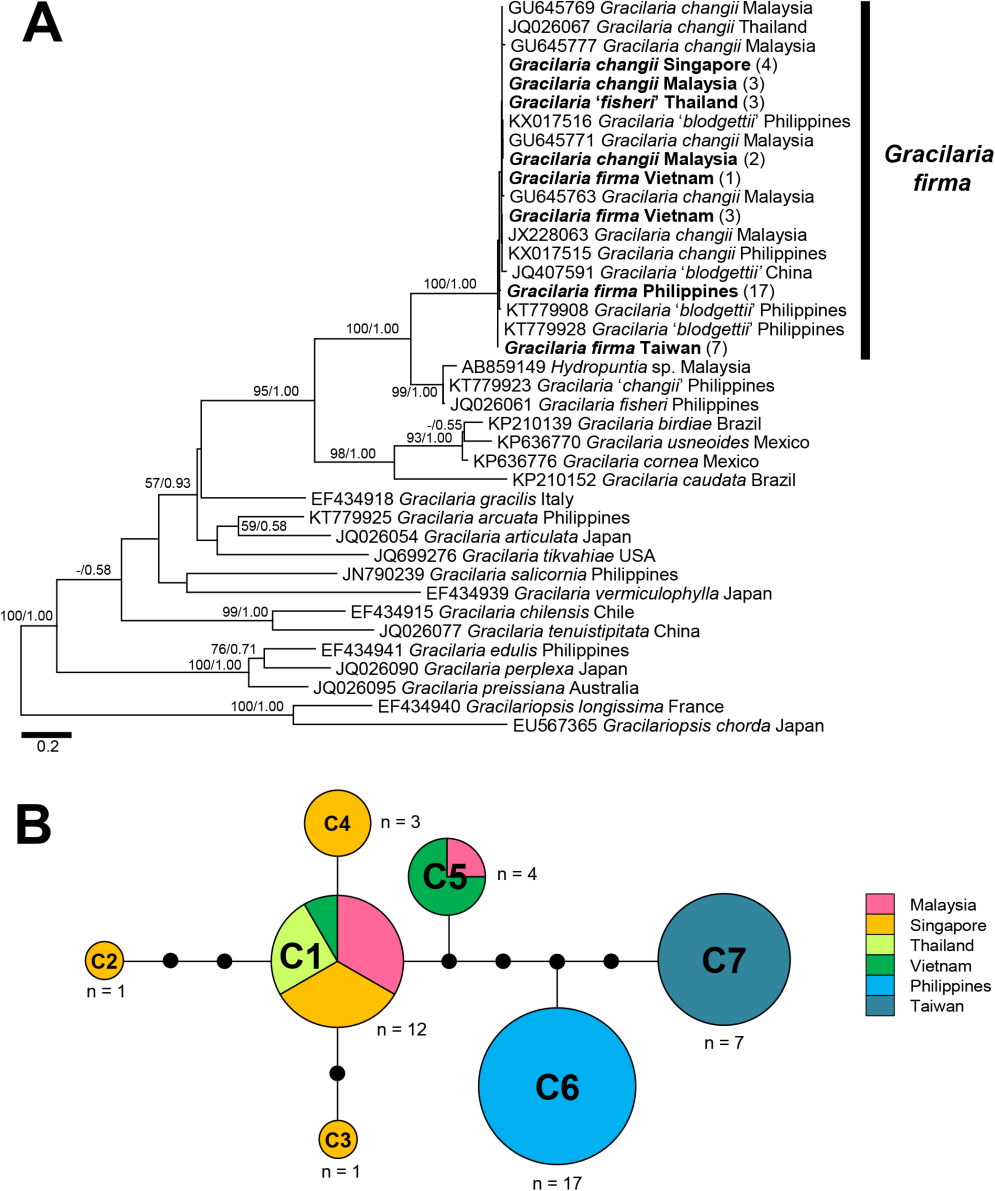
Phylogeny and haplotype network for *Gracilaria firma* based on the *cox*1 gene. **(A)** Phylogeny of *Gracilaria* inferred from the *cox*1 gene alignment consisting of 1118 base pairs (bp) using the ML method (-lnL = 5827.26951). Numbers at the nodes denote ML bootstrap support and Bayesian posterior probabilities. Dashes denote ML bootstrap support or Bayesian posterior probability less than 50% or 0.50, respectively. Numbers in parentheses after taxa denote the number of specimen with identical sequences from each country. **(B)** Haplotype network for *G*. *firma* based on the alignment of partial *cox*1 genes consisting of 834 bp. Circle size is proportional to haplotype frequency. Lines connecting the haplotypes represent single base pair mutations. Small black circles represent undetected or hypothetical haplotypes. Haplotypes are colored according to the geographic origin as shown in the key.

***cox*1 gene**. The topologies of the phylogenies for 39 taxa inferred using ML and BI methods are largely congruent. A strongly supported group that corresponds to Clade I in the *rbc*L phylogeny was recovered in the *cox*1 phylogeny (Fig 4A). The relationships among the remaining *Gracilaria* taxa were unresolved as hard polytomies in the Bayesian tree and with very poor nodal support from both analyses. All samples sequenced in this study formed a fully supported monophyletic clade with the GenBank entries for *G*. *changii* from Malaysia, Thailand and the Philippines, along with the Chinese and Philippines *G*. ‘*blodgettii*’. These monophyletic taxa differed from each other by 0–2.4% pairwise distance over the 1118-bp alignment. These specimens were most closely related to an unnamed Malaysian ‘*Hydropuntia*’ species as well as *G*. ‘*changii*’ and *G*. ‘*fisheri*’ from the Philippines, to which they differed by 7.1–8.2%. Interspecific divergence of other samples of Atlantic origin within the *G*. *caudata* clade to the *G*. *firma/G*. *changii/G*. ‘*fisheri*’ analyzed in this study ranged from 11.7% to 16.3%.

Forty *cox*1 gene sequences generated from this study and five published sequences representing different haplotypes of *G*. *changii* [32–33] were collapsed into seven haplotypes of 834 bp with 13 variable sites that were connected by a maximum of eight mutational steps (Fig 4B, Table 2). Haplotype C1 was shared among *G*. *firma* from Vietnam, *G*. ‘*fisheri*’ from Thailand and *G*. *changii* from Malaysia and Singapore, whereas haplotype C5 was shared with the Vietnamese *G*. *firma* and Malaysian *G*. *changii*. The remaining haplotypes C2, C3 and C4 were unique to Malaysian samples, each differing from haplotype C1 by one to three mutational steps. The haplotypes C6 and C7, each representative of *G*. *firma* from the Philippines and Taiwan, were more closely related to one another than they were to the haplotypes C1-C5 that represent the mainland-bound countries. The former differed from one another by three mutational steps, while they differed from the latter by five to eight mutational steps (Fig 4B, Table 2). The overall haplotype and nucleotide diversities of the populations examined in this study based on *cox*1 were 0.766 ± 0.038 and 0.00364 ± 0.00024, respectively. In contrast to the populations from Thailand, Singapore, the Philippines and Taiwan that showed no variation, the samples from Malaysia and Vietnam recorded a haplotype diversity of 0.800 and 0.500, and nucleotide diversity of 0.00224 and 0.00120, respectively.

**Table 2:** Variation sites in DNA sequences of *Gracilaria firma* for haplotypes based on the *cox*1 gene.

Haplotypes	Variation sites in 834-bp alignment												
	25	107	171	262	358	409	415	518	590	643	727	757	784
C1	A	C	A	G	C	T	T	C	G	A	A	T	T
C2	A	C	C	G	C	C	T	C	G	A	G	T	T
C3	C	T	A	G	C	T	T	C	G	A	A	T	T
C4	A	C	A	G	C	T	T	C	G	G	A	T	T
C5	A	C	A	G	T	T	T	C	A	A	A	T	T
C6	A	C	A	G	T	T	C	T	G	A	A	T	G
C7	A	C	A	A	T	T	T	T	G	A	A	C	G

### Morphological and anatomical observations

**Fig 5:**
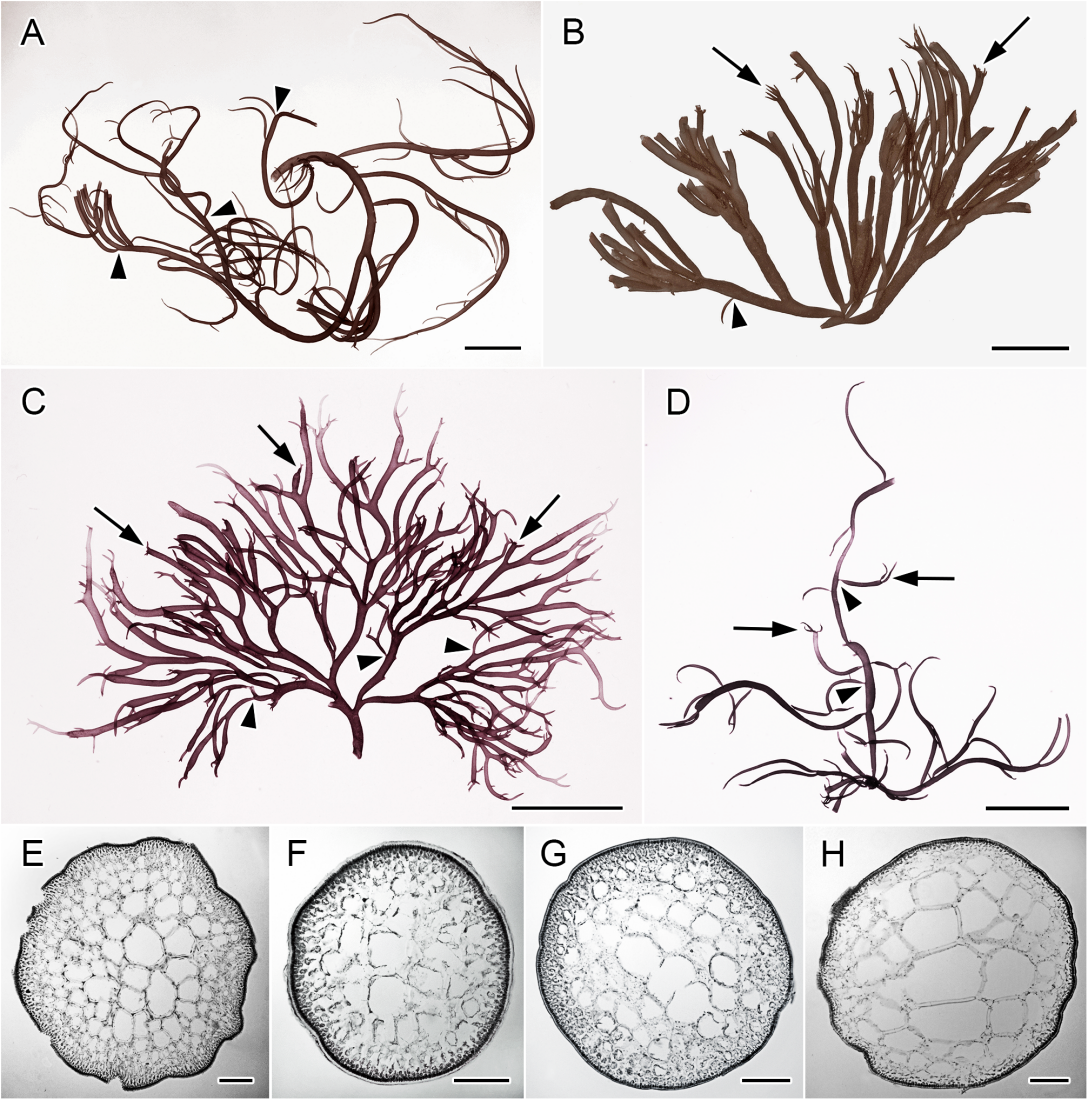
Thalli habit and cross sections of liquid-preserved *Gracilaria firma* from different localities. Note the common basal constrictions (arrowheads) of branches that taper to either acute apices or truncated tips that tend to regenerate new branchlets (arrows). **(A)** Tetrasporic plant from the Philippines (#00013). Scale bar = 2 cm. **(B)** Another tetrasporic plant with thick axes and dense branching from the Philippines (#00045). Scale bar = 2 cm. **(C)** Sterile plant from a cultivation farm in Taiwan showing intense branching with many newly regenerated branchlets (NTOU-KH-5i2016-Gf). Scale bar = 2 cm. **(D)** Sterile plant from a wild population in Malaysia as *G*. *changii* showing several branches with less robust branching arising from a single holdfast (PSM12881). Scale bar = 2 cm. **(E)** Transverse section through young vegetative branch of a tetrasporic plant from the Philippines (#00013). Scale bar = 0.25 mm. **(F)** Transverse section through young thallus of *G*. *firma* from Taiwan (NTOU-KH-5i2016-Gf). Scale bar = 0.25 mm. **(G)** Transverse section through young thallus of *G*. *changii* from Malaysia (PSM12881). Scale bar = 0.25 mm. **(H)** Transverse section through older thallus of *G*. *firma* from Taiwan (NTOU-KH-5i2016-Gf). The transition of cell size from the cortex to medulla is gradual in young branches and abrupt in older branches. Scale bar = 0.25 mm.

Samples from Taiwan, Vietnam and the Philippines displayed morpho-anatomical features that conformed to the circumscription for *G*. *firma* (Fig 5). Their identity was also confirmed by morphological comparison with the holotype and isotype of *G*. *firma* (Fig 2A and 2B). Samples from Malaysia and Singapore were identified as *G*. *changii* based on geographical provenance, morphological comparison with the holotype, and molecular evidence. Although our sterile *G*. *changii* collections precluded anatomical observation of the reproductive structures, they showed identical *rbc*L and *cox*1 sequences as those collected from the type locality in Penang, Malaysia. The voucher samples of the Thai samples were lost and not morphologically examined; therefore, they were only provisionally assigned as *G*. ‘*fisheri*’ based on their geographical provenance. In addition to the molecular data, careful comparison of the morphological observations of type specimens (Fig 2) and the species descriptions [3, 5] (Table 3) revealed that *G*. *firma* and *G*. *changii* showed no inherent differences to support their treatment as distinct species. In accordance with the principle of priority in nomenclature, we propose to reduce *G*. *changii* to *G*. *firma*, and the morpho-anatomical descriptions are given below.

**Table 3:** Morphological comparison of *Gracilaria firma*, *G*. *changii* and *G*. *fisheri*.

	*Gracilaria firma* Chang & Xia	*Gracilaria changii* (Xia & Abbott) Abbott, Zhang & Xia	*Gracilaria fisheri* (Xia & Abbott) Abbott, Zhang & Xia
Reference	[3]	[5]	[5]
Type locality	Guangdong Province, China	Pinang, Malaysia	Pattani, Thailand
Distribution	China	Philippines, Malaysia, Thailand	Thailand
Thallus habit	Erect, 10–20 cm in length	Erect and turgid, 6–20 cm in length	Erect, 15–30 cm in length
Branching pattern	Alternate or secund	Irregular, alternate or secund	Alternate, occasionally secund
Branch diameter	1–2 mm, up to 3 mm	0.3–1.5 mm, up to 2 mm	0.5–2 mm
Cell size gradation	Gradual, with medulla cells measuring 230–360 μm, up to 450 μm	Abrupt, with medulla cells measuring 350–570 μm	Gradual, with medulla cells measuring 240–560 μm
Cystocarp shape and size	Conical or hemispherical, 580–630 μm in diameter	Conical or semiglobose, up to 550 μm in diameter, slightly rostrate, not constricted at the base	Conical or semiglobose, rostrate, up to 700 μm in diameter, not constricted at the base
Nutritive filaments	Absent between gonimoblasts and pericarp, but restricted to cystocarp floor	Present scarcely over the cavity	Few present over the cavity
Pericarp	8–10 layers of cells, of which the first two layers of outermost cells are roundish square to oblong, inner layers of cells are horizontally oblong, and cells in the innermost layers are elliptical to roundish; 83–95 μm thick	Composed of two types of tissues, with 5–6 rows of rounded to oval cells on the outer layer and 7–9 rows of horizontally oblong cells on the inner layer	Composed of horizontally compressed cells with obscure cell walls and star-shaped contents
Gonimoblast	Small, densely massed cells with thicker walls	Small cells	Small cells
Spermatangial conceptacles	Elliptical cavities measuring 66–116 × 33–66 μm (*verrucosa*-type)	Oval to nearly globose cavities confluent into irregularly shaped cavity; individual conceptacle measuring 25–87 × 15–33 μm (*Polycavernosa*-type)	Globular to irregular, compound when mature; individual conceptacle measuring 40–63 × 43–50 μm (*Polycavernosa-*type)

#### *Gracilaria firma* Chang & Xia. Specimens examined

(1) Philippines, Sorsogon, Bulusan: AQ Hurtado, 4 February 2015, #00012 and #00045 (tetrasporic); #00011, #00013, #00014 and #00016 (males); #00041-#00044 (females) (2) Taiwan, Yunlin County: LC Liu, 5 January 2016, NTOU-KH-5i2016-Gf (sterile) (3) Malaysia, Selangor, Morib: PK Ng, 14 December 2015, PSM12881 (sterile).

#### Habit and vegetative structure

Plants are erect and measured up to 20 cm in length. Several main axes of 1-2(-4) mm in diameter arise from a small discoid holdfast. Thalli are densely (Fig 5A–5C) to scarcely branched (Fig 5D). The branching is mainly irregular, sometimes alternate or secund in portions, and up to four orders or more. Branches are terete, always constricted at the base, either only slightly or deeply (Fig 5A–5D, arrowheads), and taper gradually toward acute or obtuse apices. Obtuse apices occur when thallus tips are broken, and two or more fine, short branchlets tend to regenerate from a single broken tip (Fig 5B–5D, arrows). Thalli texture is firm and cartilaginous. Color ranges from light or brownish green to olive black when fresh.

Transition of cell size from cortex to medulla is gradual in the thalli of higher branching orders (Fig 5E–5G) and abrupt in the primary or secondary branches (Fig 5H). The thallus consists of one to two layers of pigmented cortical cells measuring 7.5–12.5 μm long by 3.8–7.5 μm wide, two to three layers of subcortical cells measuring 47.5–87.5 μm long by 25–40 μm wide, and five to nine layers of lightly staining medullary cells, which are irregularly subspherical to polygonal, measuring from 60–100 μm to 180–250 μm in diameter, and up to 530 μm in older thalli (Fig 5H).

#### Reproductive structures

**Fig 6:**
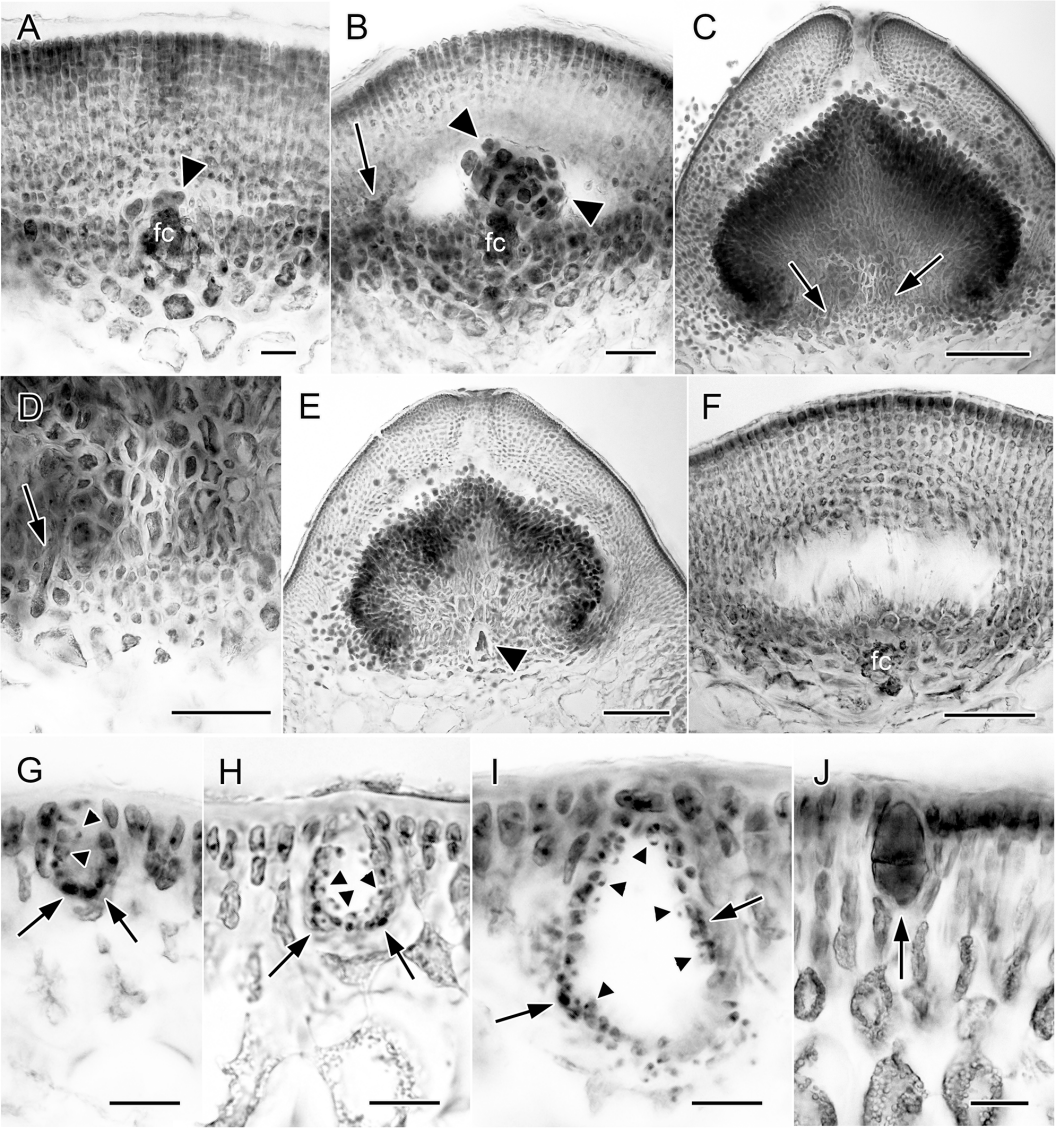
Reproductive morphology of *Gracilaria firma* from the Philippines. **(A)** Early stage of cystocarp development showing fusion cell (fc) and gonimoblast initial (arrowhead). Scale bar = 25 μm. **(B)** Young cystocarp section showing the fusion cell (fc) bearing gonimoblast cell clusters (arrowheads). Note the development of multinucleate tubular nutritive cells at the inner pericarp (arrow). Scale bar = 50 μm. **(C)** Mature cystocarp section showing multinucleate tubular nutritive cells (arrows) distributed around the floor. Scale bar = 200 μm. **(D)** Close-up of (C) showing the inconspicuous tubular nutritive cells (arrow) connecting the gonimoblast cells and inner pericarp cells at the floor. Scale bar = 100 μm. **(E)** Mature cystocarp showing a persistent fusion cell (arrowhead) and tightly packed small gonimoblast cells terminally bearing carpospores in straight chains. Scale bar = 200 μm. **(F)** Aborted cystocarp where the fusion cell (fc) has failed to produce gonimoblasts. Scale bar = 100 μm. **(G)** A young spermatangial conceptacle showing spermatangial parent cells (arrows) and spermatangia (arrowheads). Scale bar = 20 μm. **(H)** A developing spermatangial conceptacle showing spermatangial parent cells (arrows) and spermatangia (arrowheads). Scale bar = 20 μm. **(I)** Mature spermatangial conceptacle of the *verrucosa*-type lined by spermatangia (arrowheads) cut off from spermatangial parent cells (arrows). Scale bar = 20 μm. **(J)** An immature tetrasporangium embedded in the cortical layer of elongated cells showing pit-connection (arrowhead) to a subcortical cell. Scale bar = 20 μm.

The gametophytes are dioecious. Tetrasporophytes and gametophytes are isomorphic. Cystocarps are borne over the surfaces of the fertile thalli. Mature cystocarps are hemispherical and slightly or not constricted at the bases and 0.8–1.1(-1.8) mm in diameter. Carpogonial branches and early postfertilization stages were not seen in the specimens examined. After presumed fertilization, fusion cells (Fig 6A, arrowhead) begin to form by the progressive incorporation of sterile branches and neighboring vegetative cells to the cells of the carpogonial branch. The formation of the pericarp and fusion cell is more or less complete prior to gonimoblast initiation. As the gonimoblasts develop, the pit connections between the pericarp cells at the level of the fusion cell break down to initiate a schizogenous cavity (Fig 6A), which enlarges as the pericarp becomes thicker, and the gonimoblasts grow (Fig 6B). The cells at the cystocarp floor or inner pericarp start to form multinucleate tubular nutritive cells that extend to the lower part of the outer pericarp (Fig 6B, arrow). At maturity, the innermost cells of gonimoblasts are vacuolated and united by numerous secondary pit connections, whereas the outer gonimoblast cells differentiate into tightly packed small cells bearing short straight chains of carposporangia (Fig 6C). Gonimoblast cells at the cystocarp floor pit-connect to the inner pericarp cells secondarily. Inconspicuous multinucleate traversing filaments connecting only the inner pericarp cells and gonimoblast cells are formed at the cystocarp floor (Fig 6C–6E, arrows). The fusion cell is persistent in mature cystocarp (Fig 6E, arrowhead). Mature pericarps are 8–14 cell layers thick. Carposporangia are ovoid or obovoid, 19–31 μm long by 15–22 μm wide (Fig 6E). The cystocarp sometimes fails to develop beyond the fusion cell-stage and generate gonimoblasts, but the pericarp continues to enlarge as in developing cystocarps (Fig 6F). Thalli with aborted carposporophytes also bear cystocarps of normal size and form. Reproductive structures are scattered over the thallus except the basal and extreme apical portions. Spermatangial parental cells are initiated from outer cortical cells and are arranged in depressions that form the spermatangial conceptacles (Fig 6G). Mature spermatangia are 3–5 μm long by 1.5–2.3 μm wide, scattered over the surface of the deep *verrucosa*-type conceptacles with an average depth of 50 μm up to 120 μm (Fig 6H–6I). Tetrasporangia are initiated superficially from terminal cells cut off by oblique, longitudinal cell division of the outer cortical cells. They enlarge and are surrounded by elongated cortical cells (Fig 6J). Mature tetrasporangia measure up to 40 μm long by 20 μm wide and divide twice to produce four cruciately arranged tetraspores.

## Discussion

### Conspecificity of *Gracilaria firma* and *G. changii*

Many *Gracilaria* species with stipitate branches that taper toward acute apices have been described from the western Pacific, including *G*. *firma*, *G*. *changii* and *G*. *fisheri* (Fig 2). They were likened to *G*. *blodgettii* from the western Atlantic based on their superficial branch morphology [3, 36]. The Atlantic entity was distinguished from its Pacific counterparts by the shallow spermatangial conceptacles of the *textorii*-type [5, 37]. *Gracilaria firma*, *G*. *changii* and *G*. *fisheri* were traditionally distinguished by differences in the thallus size, cell size transition from cortex to medulla, presence or absence of the nutritive filaments in the cystocarp cavity, as well as the shape of spermatangial conceptacles (Table 3).

Molecular analyses based on the *rbc*L and *cox*1 gene sequences showed that specimens identified as *G*. *firma*, *G*. *changii* and *G*. ‘*fisheri*’ are very similar. The specimens examined in this study also formed a monophyletic clade with several unpublished GenBank entries for *G*. ‘*blodgettii*’ from China and the Philippines with full nodal support in the phylogenetic reconstructions (Figs 3A and 4A). Taxa in the monophyletic clade were most closely related to an unnamed *Hydropuntia* species from Malaysia as well as some sequences identified as *G*. *changii* and *G*. *fisheri* from the Philippines, of which they differed by 4.0–4.8% over 1070 bp of the *rbc*L and 7.1–8.2% over 1118 bp of the *cox*1 genes. The nucleotide divergence based on *rbc*L was considered well beyond the intraspecific threshold of 1.5–2.0% recommended for members of Gracilariaceae [26]. In addition, *G*. ‘*changii*’ and *G*. ‘*fisheri*’ from the Philippines were not sampled from the type locality and thus their actual identity remained unverified. On the other hand, our specimens included samples of *G*. *firma* identified after thorough morpho-anatomical examination, as well as *G*. *changii* collected from the type locality in Malaysia, of which all formed a monophyletic clade in the phylogenies inferred using both *rbc*L and *cox*1 genes. These taxa showed nucleotide divergence of 0.6% and 2.4% in the *rbc*L and *cox*1 gene sequences, respectively, which are below the intraspecific nucleotide variation threshold of 1.5% and 2.6% suggested for the *rbc*L [26] and *cox*1 [38] genes, respectively, in Gracilariaceae. This implied that specimens passing under the different names of *G*. *firma*, *G*. *changii*, *G*. ‘*fisheri*’ and *G*. ‘*blodgettii*’ in the western Pacific, all characterized by the presence of stipitate branches tapering toward acute apices, may belong to a single genetic entity (Figs 3A and 4A). We were unable to examine the morpho-anatomical features of the samples provisionally designated as *G*. ‘*fisheri*’ from Thailand analyzed in this study, as the vouchers were lost. Nevertheless, the results suggested the conspecificity of *G*. *firma* and *G*. *changii*. We did not consider the conspecificity of *G*. *fisheri* with *G*. *firma*, since the actual identity of the Thai specimens as *G*. ‘*fisheri*’ cannot be ascertained by morphological observations in this study.

Our recent collections exhibit similar morphology to the type specimens (Fig 2) and the species descriptions in the literature (Table 3). The *rbc*L and *cox*1 gene sequence analyses also support the treatment of *G*. *firma* and *G*. *changii* as a single species. Although the specimens of G. *firma* examined in this study are not from the type locality, species identification was confirmed by comparing the morpho-anatomical observations with previous literature [3, 11–12] and type specimens of *G*. *firma* (Fig 2A and 2B). Specimens from Vietnam, Taiwan and the Philippines typically exhibited gradual transition in cell size from cortex to medulla, cystocarps with small gonimoblast cells and spermatangial conceptacles of the *verrucosa*-type, all characteristic of *G*. *firma* (Table 3).

The type of transition in cell size from cortex to medulla, whether gradual or abrupt, was considered to be consistent within *Gracilaria* species [39]. However, we observed that the specimens of *G*. *firma* from Taiwan showed gradual transition in cell size from cortex to medulla in young branches (Fig 5E–5G) and abrupt cell size transition in mature branches (Fig 5H). A continuum in the type of cell size transition across vegetative thallus of different ages was also observed in *G*. *blodgettii* [37]. The gradation of cell size across the thallus appears to vary with age and may not be a diagnostic taxonomy character. The nutritive filaments are a prominent feature of *Gracilaria sensu lato* and the position of these filaments varies in species. Most of the members of *Gracilaria sensu stricto* have the nutritive filaments extending from gonimoblast cells to both the outer pericarp and the cystocarp floor, while these filaments are restricted to the cystocarp floor in *Hydropuntia* [30, 40]. However, several detailed studies on the cystocarpic development (with up to 50 cystocarps examined) showed that some species of *Gracilaria sensu stricto*, including *G*. *huangii* S.-M. Lin & De Clerck [41] and *G*. *taiwanensis* S.-M. Lin, L.-C. Liu & Payri [18], have nutritive filaments restricted to the cystocarp floor and a virtual lack of these filaments between the gonimoblasts and outer pericarp. It should be noted that *G*. *firma* was originally described to be lacking nutritive filaments between the gonimoblasts and pericarp (page 144, lines 3–4) [3]. As mentioned earlier, nutritive filaments do occur at the cystocarp floor in many species of *Gracilaria*, including species which are also lacking the filaments between pericarp and gonimoblasts. Detailed morpho-anatomical observations on the specimens of *G*. *firma* from our sequenced collection revealed the existence of nutritive filaments that are only weakly formed at the cystocarp floor (Fig 6C and 6D). We speculated that such nutritive filaments might have been overlooked when *G*. *firma* was first described, as the filaments are not as conspicuous as those present in other members of *Gracilaria*. The presence of such inconspicuous nutritive filaments connecting the gonimoblasts and cystocarp floor in *G*. *firma* echoed the scarce presence of basal absorbing filaments in *G*. *changii* [5]. The type of spermatangial conceptacles was considered as the main differentiating feature that set *G*. *firma* and *G*. *changii* apart [13], but a mixture of spermatangial conceptacles of the *verrucosa*-type and *Polycavernosa-*type had been observed within the same branchlet of *G*. *changii* and *G*. *fisheri* [2]. As such, there seems to be no absolute designation of male type in a single species, at least in *G*. *changii* and *G*. *fisheri*, where spermatangial conceptacles of the *verrucosa*-type and *Polycavernosa*-type can grade into each other. The *Polycavernosa*-type had been considered as a variation, and sometimes the mature manifestations, of the *verrucosa*-type of spermatangial conceptacles [2]. Our detailed morpho-anatomical observations, as well as a comparison of the species description in literature, showed that the reproductive anatomy of *G*. *firma* is largely congruent with that circumscribed for *G*. *changii*. Both *G*. *firma* and *G*. *changii* are characterized by branches that are constricted at the base and taper toward acute or obtuse (broken) apices, cystocarps consisted of small gonimoblast cells with carposporangia borne at the periphery and inconspicuous multinucleate tubular nutritive cells extending from gonimoblasts at the cystocarp floor, as well as spermatangia borne in deep conceptacles of *verrucosa*-type.

Based on the molecular data and morphological observations, we recommended the merging of *G*. *changii* and *G*. *firma*, with the oldest validly published name, *G*. *firma* Chang & Xia, being adopted for this genetic species according to the principle of priority in nomenclature. Accordingly, the species with the aforementioned morpho-anatomical features from the western Pacific (i.e., *G*. *changii*) should be treated as *G*. *firma*, and this taxonomic conclusion is made formal below. The taxonomy status of *G*. *fisheri* will be a topic for future work until more samples are available for examination.

### Genetic diversity of *Gracilaria firma*

The specimens examined in this study exhibited morphological variations in branch size and branching pattern for the five chlorotypes (R1-R5) and mitotypes (C1, C4-C7) recovered. Some bushy specimens with more than four branching orders display very short internodes with many branches and branchlets, whereas others with a branching order of three or less can have very few or many unilateral branchlets. Occasionally, two thick branches can coalesce into one, of which the medullary tissues had degenerated and left behind a hollow cavity.

The genetic diversity estimates for *G*. *firma* were similar to those reported for *Gracilariopsis chorda* (Holmes) Ohmi [42] and *G*. *vermiculophylla* (Ohmi) Papenfuss [43] in the northwestern Pacific, with high haplotype (~0.7) but low nucleotide diversities (~0.002–0.003). *Gracilaria firma* showed apparent phylogeographic structure across the countries bordering the South China Sea, which is likely to be shaped by the oceanographic conditions in the western Pacific. The recovery of a Vietnamese sample with identical haplotypes association as the samples from Malaysia, Singapore and Thailand (R1-C1) implied genetic connectivity along the coastline of the Southeast Asian mainland (S1 Table, Fig 1). The haplotype C5, deemed unique to the remaining Vietnamese samples examined in this study, is apparently identical to a GenBank entry of *G*. *changii* (JX228063) collected from the east coast of Peninsular Malaysia over 834 bp (Fig 4B). Genetic homogeneity of the *G*. *firma* populations from the mainland-bound countries could be a result of postglacial recolonization. These countries were once connected as part of the Sunda shelf which emerged with other land bridges during the Pleistocene [44]. The South China Sea that is enclosed by the Sunda Shelf may have served as one of the refugia where population isolation and differentiation occurred [45] to produce the rather high number of haplotypes recovered in the *G*. *firma* sampled along Vietnam to the Malay Peninsula (three chlorotypes, R1-R3 and five mitotypes, C1-C5). The Monsoon-driven current circulation pattern of the South China Sea and Malacca Strait may have promoted spore dispersal, thus maintaining gene flow in the populations along the coastlines of Vietnam, Malaysia and Singapore. Populations in the Philippines (R4-C6) and Taiwan (R5-C7) appeared to be genetically disconnected from the populations in the mainland-bound countries. The San Bernardino Strait that carries water from the western Pacific into the Sulu Sea may have limited the gene flow from Sorsogon in the Philippines with the Malaysian, Singaporean, Thai and Vietnamese populations that are in direct contact with the South China Sea (Fig 1), thus maintaining its unique haplotype. Several studies revealed that red algal species such as *Gracilaria salicornia* (C. Agardh) Dawson [46] and *Gelidiella fanii* S.-M. Lin [47] also showed divergence between populations from the Philippines and other mainland-bound Southeast Asian countries. Similarly, maintenance of the endemic haplotype in Taiwan could be attributed to the unidirectional northeastward circulation pattern of the Kuroshio Current that flows from the Pacific across the Luzon Strait into the Taiwan Strait, thereby restricting the gene flow between the Taiwanese population and the Southeast Asian populations (Figs 1, 3B and 4B). Cultivation of the agarophyte by means of vegetative propagation could also contribute to fixation of particular haplotypes at a regional scale [48]. The possibility of anthropogenic factors mediating the genetic connectivity of *G*. *firma* around South China Sea cannot be ruled out as the shipping traffic and material exchange have not been well documented. However, this region appeared to be the native distribution range of this species and probabilities are high that farms have been established only using local material.

The establishment of molecular markers as barcodes for rapid species identification to drive further multidisciplinary studies is only meaningful when a species is correctly assigned a validly published name to represent a single genetic species. A significant correlation between haplotype richness and the number of collection sites had been reported [49]. Intensive sampling effort to include samples from multiple distinct sites is necessary to reveal the genetic diversity of the species in the region more comprehensively. This is especially relevant in the context of agarophyte cultivation, as limited genetic diversity of crops jeopardizes the potential for sustained genetic improvement over the long term and renders them more susceptible to disease and pest epidemics [48, 50].

### Taxonomic conclusion

*Gracilaria firma* Chang & Xia [Stud Mar Sinica. 1976;11:143, figs 38–39; 162, Plate II fig 4] *sensu emend*. P.-K. Ng, S.-M. Lin, P.-E. Lim, A.Q. Hurtado, S.-M. Phang, Y.-Y. Yow et al.

Holotype: AST 66–450 (Allotype: AST 66-540a, AST 66-540b)

Type locality: Xindi, Xuwen County, Guangdong Province, China

Proposed synonyms: *Polycavernosa changii* Xia & Abbott [Phycologia. 1987; 26:407]; *Hydropuntia changii* (Xia & Abbott) Wynne [Taxon. 1989; 38:476]; *Gracilaria changii* (Xia & Abbott) Abbott, Zhang & Xia [Pac Sci. 1991;45(1):23] [Holotype: Male and tetrasporic, Fisher #1022 in the Herbarium of B.P. Bishop Museum, Honolulu, Hawaii (BISH); type locality: intertidal mud flat approximately 200 m northeast of beacon opposite Glugor Marine Police jetty, attached to edge of old coconut shell partly buried in mud, Middle Bank, Pinang, Malaysia, 10 March 1983].

Distribution: Widely distributed in the tropical and subtropical regions in the western Pacific, including China [3, 7, 51–52], Malaysia (as *G*. *firma* and *G*. *changii* in [7, 16]; *G*. *changii* in [5, 32–33]; and *G*. *firma* in [53]), the Philippines (as *G*. *firma* and *G*. *changii* in [54]; *G*. *firma* in [7]), Thailand (as *G*. *firma* and *G*. *changii* in [7, 55]; *G*. *changii* in [15]), Vietnam (as *G*. *firma* in [13]; *G*. *verrucosa*, *G*. *fisheri* and *G*. *blodgettii* in [11]), Japan [56], Taiwan (as *G*. *confervoides* in [57]; *G*. *verrucosa* in [58]; *G*. *blodgettii* in [59]; G. *tenuistipitata* var. *liui* [8]; and *G*. *firma* in [12])

## Supporting information

S1 Table Collection information and GenBank accession numbers for the *rbc*L and *cox*1 gene sequences of the specimens examined in this study. Voucher number in boldface indicates sample used for morphological analyses; asterisk after voucher number indicates sample collected from type locality.
(DOCX)
