# Supplementary material for: Genetic and morphological analyses of Gracilaria firma and G. changii (Gracilariaceae, Rhodophyta), the commercially important agarophytes in western Pacific
Source: PLoS One. 2017 Jul 31;12(7):e0182176. doi: 10.1371/journal.pone.0182176 (PMC5536327; doi:10.1371/journal.pone.0182176)
Supplement: S1 Table — Voucher number in boldface indicates sample used for morphological analyses; asterisk after voucher number indicates sample collected from type locality. (DOCX) [file pone.0182176.s001.docx]

**S1 Table. Collection information and GenBank accession numbers for the *rbc*L and *cox*1 gene sequence of the specimens examined in this study.** Voucher number in boldface indicates sample used for morphological analyses; asterisk after voucher number indicates sample collected from type locality.

| Taxon | Isolate | Locality | Voucher number | Collection date | GenBank accession number; haplotype | |
| --- | --- | --- | --- | --- | --- | --- |
|  |  |  |  |  | *rbc*L | *cox*1 |
| *Gracilaria firma* Chang et Xia | TW3 | Taiwan: Kouhu Township, Yunlin County | NTOU-KH-20viii2015-3 | 20 Aug 2015 | KY315286; R5 | KY315246; C7 |
|  | TW7 | Taiwan: Kouhu Township, Yunlin County | NTOU-KH-20viii2015-7 | 20 Aug 2015 | KY315287; R5 | KY315247; C7 |
|  | TW14 | Taiwan: Kouhu Township, Yunlin County | NTOU-KH-20viii2015-14 | 20 Aug 2015 | KY315288; R5 | KY315248; C7 |
|  | TW18 | Taiwan: Kouhu Township, Yunlin County | NTOU-KH-20viii2015-18 | 20 Aug 2015 | KY315289; R5 | KY315249; C7 |
|  | TW20 | Taiwan: Kouhu Township, Yunlin County | NTOU-KH-20viii2015-20 | 20 Aug 2015 | KY315290; R5 | KY315250; C7 |
|  | TW23 | Taiwan: Kouhu Township, Yunlin County | NTOU-KH-20viii2015-23 | 20 Aug 2015 | KY315291; R5 | KY315251; C7 |
|  | TW35 | Taiwan: Kouhu Township, Yunlin County | NTOU-KH-20viii2015-35 | 20 Aug 2015 | KY315292; R5 | KY315252; C7 |
|  | - | Taiwan: Kouhu Township, Yunlin County | **NTOU-KH-5i2016-Gf** | 5 Jan 2016 | - | - |
|  | Gsp1 | Taiwan: Tungkang Fishery Institute, Pingtung County | NTOU-TK-7ix2005-1 | 7 Sept 2005 | KY315326; R5 | - |
|  | Gsp2 | Taiwan: Tungkang Fishery Institute, Pingtung County | NTOU-TK-7ix2005-2 | 7 Sept 2005 | KY315327; R5 | - |
|  | Gsp3 | Taiwan: Tungkang Fishery Institute, Pingtung County | NTOU-TK-7ix2005-3 | 7 Sept 2005 | KY315328; R5 | - |
|  | Gsp4 | Taiwan: Tungkang Fishery Institute, Pingtung County | NTOU-TK-7ix2005-4 | 7 Sept 2005 | KY315329; R5 | - |
|  | V2 | Vietnam: Thi Nai Lagoon, Qui Nhon, Qinh Dinh | - | 1 Apr 2005 | KY315293; R3 | KY315253; C5 |
|  | V3 | Vietnam: Thi Nai Lagoon, Qui Nhon, Qinh Dinh | - | 1 Apr 2005 | KY315294; R3 | KY315254; C5 |
|  | V5 | Vietnam: Cat Hai, Hai Phong | - | 19 Apr 2003 | KY315295; R3 | KY315255; C5 |
|  | V10 | Vietnam: Ham Ninh, Phu Quoc Island | - | 20 Sept 2003 | KY315296; R1 | KY315256; C1 |
|  | A8 | Philippines: Bulusan, Sorgoson | **#00008** | 4 Feb 2015 | KY315297; R4 | KY315257; C6 |
|  | A9 | Philippines: Bulusan, Sorgoson | **#00009** | 4 Feb 2015 | KY315298; R4 | KY315258; C6 |
|  | A10 | Philippines: Bulusan, Sorgoson | **#00010** | 4 Feb 2015 | KY315299; R4 | KY315259; C6 |
|  | A11 | Philippines: Bulusan, Sorgoson | **#00011** | 4 Feb 2015 | KY315300; R4 | KY315260; C6 |
|  | A12 | Philippines: Bulusan, Sorgoson | **#00012** | 4 Feb 2015 | KY315301; R4 | KY315261; C6 |
|  | A13 | Philippines: Bulusan, Sorgoson | **#00013** | 4 Feb 2015 | KY315302; R4 | KY315262; C6 |
|  | A15 | Philippines: Bulusan, Sorgoson | **#00015** | 4 Feb 2015 | KY315303; R4 | KY315263; C6 |
|  | A16 | Philippines: Bulusan, Sorgoson | **#00016** | 4 Feb 2015 | KY315304; R4 | KY315264; C6 |
|  | A41 | Philippines: Bulusan, Sorgoson | **#00041** | 4 Feb 2015 | KY315305; R4 | KY315265; C6 |
|  | A42 | Philippines: Bulusan, Sorgoson | **#00042** | 4 Feb 2015 | KY315306; R4 | KY315266; C6 |
|  | A43 | Philippines: Bulusan, Sorgoson | **#00043** | 4 Feb 2015 | KY315307; R4 | KY315267; C6 |
|  | A44 | Philippines: Bulusan, Sorgoson | **#00044** | 4 Feb 2015 | KY315308; R4 | KY315268; C6 |
|  | A45 | Philippines: Bulusan, Sorgoson | **#00045** | 4 Feb 2015 | KY315309; R4 | KY315269; C6 |
|  | A46 | Philippines: Bulusan, Sorgoson | **#00046** | 4 Feb 2015 | KY315310; R4 | KY315270; C6 |
|  | A47 | Philippines: Bulusan, Sorgoson | **#00047** | 4 Feb 2015 | KY315311; R4 | KY315271; C6 |
|  | A48 | Philippines: Bulusan, Sorgoson | **#00048** | 4 Feb 2015 | KY315312; R4 | KY315272; C6 |
|  | A49 | Philippines: Bulusan, Sorgoson | **#00049** | 4 Feb 2015 | KY315313; R4 | KY315273; C6 |
| *Gracilaria fisheri* (Xia et Abbott) Abbott, Zhang et Xia | K2 | Thailand: Klongwan Fisheries Research Station, Prachuap Khirikhan Province | KW2 | - | KY315314; R1 | KY315274; C1 |
|  | K4 | Thailand: Klongwan Fisheries Research Station, Prachuap Khirikhan Province | KW4 | - | KY315315; R1 | KY315275; C1 |
|  | K5 | Thailand: Klongwan Fisheries Research Station, Prachuap Khirikhan Province | KW5 | - | KY315316; R1 | KY315276; C1 |
| *Gracilaria changii* (Xia et Abbott) Abbott, Zhang et Xia | M1 | Malaysia: Sungai Boh, Johor | PSM10466 | 24 Aug 2009 | KY315317; R1 | KY315277; C1 |
|  | M4 | Malaysia: Pulau Korea Besar, Penang | PSM10481* | 10 Dec 2007 | KY315318; R1 | KY315278; C1 |
|  | M5 | Malaysia: Pulau Korea Besar, Penang | PSM11942* | 20 Mar 2011 | KY315319; R1 | KY315279; C1 |
|  | M6 | Malaysia: Kuala Kubang Badak, Kedah | PSM11949 | 21 Mar 2011 | KY315320; R1 | KY315280; C4 |
|  | M7 | Malaysia: Morib, Selangor | **PSM12881** | 14 Dec 2015 | KY315321; R1 | KY315281; C4 |
|  | G4 | Singapore: OBS camp 1, Ubin Island | JS3295 | 30 Oct 2012 | KY315322; R2 | KY315282; C1 |
|  | G5 | Singapore: OBS camp 1, Ubin Island | JS3297 | 30 Oct 2012 | KY315323; R1 | KY315283; C1 |
|  | G12 | Singapore: OBS camp 1, Ubin Island | JS3296 | 30 Oct 2012 | KY315324; R2 | KY315284; C1 |
|  | G18 | Singapore: OBS camp 1, Ubin Island | JS3284 | 30 Oct 2012 | KY315325; R1 | KY315285; C1 |
